# Exploring adverse events of Vilazodone: evidence from the FAERS database

**DOI:** 10.1186/s12888-024-05813-0

**Published:** 2024-05-16

**Authors:** Ying Jiang, Yucai Qu, Zhiqiang Du, Mengmeng Ou, Yuan Shen, Qin Zhou, Lin Tian, Haohao Zhu

**Affiliations:** Mental Health Center of Jiangnan University, Central Rehabilitation Hospital, Wuxi, Jiangsu 214151 China

**Keywords:** Vilazodone, Major depressive disorder, Adverse events, Safety profile, Signal mining

## Abstract

**Objective:**

This study aims to conduct an exhaustive evaluation of Vilazodone's safety in clinical application and to unearth the potential adverse event (AE) risks associated with its utilization based on FDA Adverse Event Reporting System (FAERS) database.

**Methods:**

This research employed data spanning from the first quarter of 2011 to the third quarter of 2023 from the FAERS database. Various signal detection methodologies, including the Reporting Odds Ratio (ROR), Proportional Reporting Ratio (PRR), Bayesian Confidence Propagation Neural Network (BCPNN), and Empirical Bayesian Geometric Mean (EBGM), were utilized to ascertain the correlation between Vilazodone and specific AEs.

**Results:**

The study compiled a total of 17,439,268 reports of drug AEs, out of which 5,375 were related to Vilazodone. Through signal mining, 125 Preferred Terms (PTs) encompassing 27 System Organ Classes (SOCs) were identified. The findings indicated a higher prevalence among females and patients within the 45 to 65 age bracket. The principal categories of AEs included Psychiatric disorders, Nervous system disorders, and Gastrointestinal disorders, with prevalent incidents of Diarrhoea, Nausea, and Insomnia. Moreover, the study identified robust signals of novel potential AEs, notably in areas such as sleep disturbances (Sleep paralysis, Hypnagogic hallucination, Rapid eye movements sleep abnormal, Sleep terror, Terminal insomnia, Tachyphrenia), sexual dysfunctions (Female orgasmic disorder, Orgasm abnormal, Disturbance in sexual arousal, Spontaneous penile erection, Anorgasmia, Sexual dysfunction, Ejaculation delayed), and other symptoms and injuries (Electric shock sensation, Violence-related symptom, Gun shot wound).

**Conclusion:**

Although Vilazodone presents a positive prospect in the management of MDD, the discovery of AEs linked to its use, particularly the newly identified potential risks such as sleep and sexual dysfunctions, necessitates heightened vigilance among clinicians.

## Introduction

Major Depressive Disorder (MDD) is a severe and prevalent psychological disorder with a high incidence rate of approximately 6.7%. This condition not only profoundly impacts the physical and mental health of individuals but also significantly impairs their quality of life. MDD severely affects the mental and physical well-being of about 10% of the global population. Patients often experience extreme mood fluctuations, cognitive impairments, behavioral abnormalities, and can even suffer brain function damage leading to social disorders and, in extreme cases, self-harm or suicide [1, 2].

Pharmacotherapy has been a critical component in treating MDD, with common drug categories including Selective Serotonin Reuptake Inhibitors (SSRIs) and Selective Norepinephrine Reuptake Inhibitors (SNRIs). These are often complemented by psychological and physical therapies as part of a comprehensive intervention strategy. International treatment guidelines currently recommend SSRIs as the first-line pharmacological treatment for most MDD patients, including sertraline, escitalopram, fluoxetine, paroxetine, etc. [3, 4]. Although SSRIs can enhance synaptic plasticity, there is significant individual variation in response to these medications [5].

Vilazodone is a drug that has garnered widespread attention in this domain. Developed by Merck KgaA in Germany, Vilazodone received FDA approval in January 2011 for the treatment of MDD in adults. It represents a novel class of antidepressants, functioning both as a SSRI and a partial agonist at the 5-HT1A receptor. Vilazodone rapidly enhances the extracellular concentration of 5-HT1A, thereby exerting its antidepressant effects promptly [6].

Despite the therapeutic efficacy demonstrated by Vilazodone in treating MDD, pharmacotherapy is always accompanied by the risk of potential adverse events (AEs). Hence, this study aims to systematically mine and analyze AE signals related to Vilazodone based on the FDA Adverse Event Reporting System (FAERS) [7–10], to comprehensively evaluate its potential risks in clinical use, providing a safety reference for clinical practice.

## Methods

### Data source

The data for this study was sourced from the FAERS database. The FAERS database compiles all information on AEs and medication errors reported to the FDA. For the purposes of this study, data packages from the first quarter of 2011 to the third quarter of 2023 were downloaded. The collected data encompassed individual records, AE reports, medication usage, treatment outcomes, and reporting sources, with a specific focus on the target drug “Vilazodone”. Following the FDA-recommended method for removing duplicate reports, we select the PRIMARYID, CASEID, and FDA_DT fields from the DEMO table. We sort by CASEID, FDA_DT, and then PRIMARYID. For reports with the same CASEID, we retain the one with the largest FDA_DT value. Secondly, for reports where both CASEID and FDA_DT are the same, we retain the one with the largest PRIMARYID value. Since the first quarter of 2019, each quarterly data package has included a list of deleted reports. After data deduplication, we remove reports based on the CASEID listed in the deleted reports list.

### Data standardization

To standardize the AE data, this study utilized the terminology set of AEs from the Medical Dictionary for Regulatory Activities (MedDRA) [11]. AEs were categorized and described using the System Organ Class (SOC) and Preferred Terms (PT) from MedDRA. Additionally, the MedDRA version 26.0 software was employed for the mapping of PTs and SOCs of AEs. Moreover, this study also utilized the related terminology set from MedDRA version 26.0 for classifying and describing AEs.

### Signal detection methods

To mine signals related to Vilazodone-associated drug AEs, this study employed various signal detection methods. The primary methods included Reporting Odds Ratio (ROR) and Proportional Reporting Ratio (PRR) from the disproportionality methods, Bayesian Confidence Propagation Neural Network (BCPNN), and Empirical Bayesian Geometric Mean (EBGM) [12–15]. The ROR helps mitigate biases in events with fewer reports. The PRR stands out for its greater specificity compared to ROR. BCPNN is adept at combining and cross-validating multi-source data. The MGPS is particularly effective in identifying signals from infrequent events. This study utilizes a blend of ROR, PRR, BCPNN, and MGPS to capitalize on their individual strengths, enhancing the scope of detection and validation from diverse angles. This integrated approach aids in more accurately identifying safety signals, reducing false positives through cross-validation and refining detection of rare adverse reactions by adjusting thresholds and variance. The core algorithms of these methods are based on a 2 × 2 contingency table, used to calculate signal strength (Tables 1 and 2). A higher signal value indicates a stronger AE signal associated with Vilazodone, signifying a higher statistical association with the target AE.

**Table 1:** Four grid table

	Target AEs	Non-target AEs	Total
Vilazodone	a	b	a + b
Non- Vilazodone	c	d	c + d
Total	a + c	b + d	N = a + b + c + d

Equation: a, number of reports containing both the target drug and target adverse drug reaction; b, number of reports containing other adverse drug reaction of the target drug; c, number of reports containing the target adverse drug reaction of other drugs; d, number of reports containing other drugs and other adverse drug reactions

**Table 2:** ROR, PRR, BCPNN, and EBGM methods, formulas, and thresholds

Method	Formula	Threshold
ROR	${\text{ROR}}=\frac{\left(a/c\right)}{\left(b/d\right)}=\frac{ad}{bc}$ ${\text{SE}}({\text{lnROR}})=\sqrt{\left(\frac{1}{a}+\frac{1}{b}+\frac{1}{c}+\frac{1}{d}\right)}$ $95\mathrm{\%CI}={{\text{e}}}^{{\text{ln}}({\text{ROR}})\pm 1.96\sqrt{ \left(\frac{1}{a}+\frac{1}{b}+\frac{1}{c}+\frac{1}{d}\right)}}$	a ≥ 3 and 95% CI (lower limit) > 1
PRR	${\text{PRR}}=\frac{a/(a+b)}{c/(c+d)}$ $SE\left({\text{lnPRR}}\right)=\sqrt{ \frac{1}{a}-\frac{1}{a+b}+\frac{1}{c}-\frac{1}{{\text{c}}+{\text{d}}}}$ $95\%\mathrm C\mathrm I=e^{\text{ln}(\text{PRR})\pm1.96\sqrt{\frac1a-\frac1{a+b}+\frac1c-\frac1{\text{c}+\text{d}}}}$	a ≥ 3 and 95% CI (lower limit) > 1
BCPNN	$IC={log}_{2}\frac{p(x,y)}{p(x)p(y)}={log}_{2}\frac{a(a+b+c+d)}{(a+b)(a+c)}$ ${\text{E}}({\text{IC}})={log}_{2}\frac{(a+\gamma 11)(a+b+c+d+\alpha )(a+b+c+d+\beta )}{\left(a+b+c+d+y\right)(a+b+\alpha 1)(a+c+\beta 1)}$ $V\left(IC\right)=\frac{1}{{(ln2)}^{2}}\left\{\left[\frac{\left(a+b+c+d\right)-a+\gamma -\gamma 11}{\left(a+\gamma 11\right)\left(1+a+b+c+d+\gamma \right)}\right]+\left[\frac{\left(a+b+c+d\right)-\left(a+b\right)+\alpha -\alpha 1}{\left(a+b+\alpha 1\right)\left(1+a+b+c+d+\alpha \right)}\right]+\left[\frac{\left(a+b+c+d\right)-\left(a+c\right)+\beta -\beta 1}{\left(a+c+\beta 1\right)\left(1+a+b+c+d+\beta \right)}\right]\right\}$ $\gamma =\gamma 11\frac{(a+b+c+d+\alpha )(a+b+c+d+\beta )}{(a+b+\alpha 1)(a+c+\beta 1)}$ $IC-2SD=E\left(IC\right)-2\sqrt{V\left(IC\right)}$	IC025 > 0
EBGM	${\text{EBGM}}=\frac{a(a+b+c+d)}{(a+c)(a+b)}$ $95\mathrm{\%CI}={{\text{e}}}^{{\text{ln}}({\text{EBGM}})\pm 1.96\sqrt{ (\frac{1}{{\text{a}}}+\frac{1}{{\text{b}}}+\frac{1}{{\text{c}}}+\frac{1}{{\text{d}}})}}$	EBGM05 > 2

*Abbreviations*: *95% CI* 95% confidence interval, *N* the number of reports, *χ2* chi-squared, *IC* information component, *IC025* the lower limit of 95% CI of the IC, *E(IC)* the IC expectations, *V(IC)* the variance of IC, *EBGM* empirical Bayesian geometric mean, *EBGM05* the lower limit of 95% CI of EBGM

## Results

### Basic characteristics of AE reports

Within the FAERS database, from the first quarter of 2011 to the third quarter of 2023, a total of 17,439,268 AE reports were collected, of which 5,375 were related to Vilazodone (Table 3). Among the reports with known gender, females accounted for 65.40%, and males for 27.65%. In terms of age, the most reports were in the 45 to 65 age group, accounting for 19.81%, followed by the 18 to 45 age group, accounting for 17.49%. Reports from individuals aged 75 and above were relatively few, comprising only 3.03%. Looking at the report years, the peak was in 2013, accounting for 28.17% of the reports. Subsequently, there was a fluctuating decrease in report numbers, dropping to 3.33% by 2023. The majority of reports were submitted by consumers (59.78%), followed by physicians (22.55%). The reports were primarily from the United States, accounting for 98.36%. Reports without a specified country comprised 1.21%, with reports from Canada, India, and Germany being very low. In terms of the severity of AEs, Hospitalization—Initial or Prolonged was the most common outcome, accounting for 5.54%, followed by Death and Disability at 1.49% and 1.06%, respectively. The most common timing for AE occurrence was within 30 days of medication use, accounting for 18.81%, with a decrease in reports over time.

**Table 3:** Basic information on AEs related to vilazodone

Factors	Number of Events (%)
**Gender**	
Female	3515 (65.40)
Male	1486 (27.65)
Unknown	374 (6.96)
**Age**	
< 18	307 (5.71)
18–45	940 (17.49)
45–65	1065 (19.81)
65–75	328 (6.10)
≥ 75	163 (3.03)
Unknown	2572 (47.85)
**Reporter**	
Consumer	3213 (59.78)
Pharmacist	246 (4.58)
Physician	1212 (22.55)
Other Health Professionals	468 (8.71)
Lawyer	1 (0.02)
Unknown	235 (4.37)
**Reported Countries**	
United States	5287 (98.36)
Not Specified	65 (1.21)
Canada	11 (0.20)
India	9 (0.17)
Germany	1 (0.02)
**Report Year**	
2011	436 (8.11)
2012	602 (11.20)
2013	1514 (28.17)
2014	315 (5.86)
2015	57 (1.06)
2016	290 (5.40)
2017	343 (6.38)
2018	205 (3.81)
2019	380 (7.07)
2020	462 (8.60)
2021	336 (6.25)
2022	256 (4.76)
2023	179 (3.33)
**Serious Outcomes**	
Death	80 (1.49)
Disability	57 (1.06)
Hospitalization—Initial or Prolonged	298 (5.54)
Life-Threatening	47 (0.87)
**Adverse Event Occurrence Time—Medication Date (days)**	
0–30	1011 (18.81)
31–60	103 (1.92)
61–90	26 (0.48)
91–120	18 (0.33)
121–150	10 (0.19)
151–180	9 (0.17)
181–360	49 (0.91)
> 360	112 (2.08)

### Risk signal mining

Signal mining for AEs where Vilazodone was the primary suspected drug identified 125 PTs involving 27 SOCs. According to the number of reports, the top five SOC categories in Table 4 were Psychiatric disorders, Nervous system disorders, Gastrointestinal disorders, General disorders and administration site conditions, General disorders and administration site conditions, consistent with the drug’s label. Additionally, Reproductive system and breast disorders, Ear and labyrinth disorders had a higher incidence and signal strength, representing new potential AEs.

**Table 4:** The signal strength of AEs of vilazodone at the SOC level

System Organ Class	SOC Code	Case Reports	ROR (95% CI)	PRR (95% CI)	χ2	IC (IC025)	EBGM (EBGM05)
Psychiatric disorders	10037175	3738	5.81 (5.59–6.03)	4.59 (4.47–4.72)	11,103.86	2.20 (2.14)	4.59 (4.42)
Nervous system disorders	10029205	2300	2.05 (1.96–2.14)	1.88 (1.81–1.96)	1037.99	0.91 (0.85)	1.88 (1.80)
Gastrointestinal disorders	10017947	1981	1.67 (1.59–1.75)	1.58 (1.52–1.65)	463.23	0.66 (0.59)	1.58 (1.51)
General disorders and administration site conditions	10018065	1779	0.63 (0.60–0.66)	0.67 (0.64–0.70)	351.08	-0.58 (-0.65)	0.67 (0.64)
General disorders and administration site conditions	10022117	1732	1.10 (1.04–1.15)	1.08 (1.04–1.13)	12.87	0.12 (0.04)	1.08 (1.03)
Skin and subcutaneous tissue disorders	10040785	598	0.72 (0.66–0.78)	0.73 (0.68–0.79)	62.50	-0.45 (-0.57)	0.73 (0.67)
Investigations	10022891	542	0.61 (0.56–0.67)	0.63 (0.58–0.68)	125.95	-0.67 (-0.79)	0.63 (0.58)
Musculoskeletal and connective tissue disorders	10028395	487	0.61 (0.56–0.67)	0.62 (0.57–0.68)	116.61	-0.68 (-0.81)	0.62 (0.57)
Eye disorders	10015919	272	0.94 (0.83–1.06)	0.94 (0.83–1.06)	1.12	-0.09 (-0.27)	0.94 (0.83)
Respiratory, thoracic and mediastinal disorders	10038738	198	0.28 (0.24–0.32)	0.29 (0.25–0.33)	367.05	-1.79 (-2.00)	0.29 (0.25)
Cardiac disorders	10007541	194	0.54 (0.47–0.63)	0.55 (0.48–0.63)	73.26	-0.86 (-1.07)	0.55 (0.48)
Metabolism and nutrition disorders	10027433	171	0.55 (0.47–0.64)	0.55 (0.48–0.64)	63.39	-0.85 (-1.07)	0.55 (0.48)
Reproductive system and breast disorders	10038604	157	1.20 (1.02–1.40)	1.20 (1.02–1.40)	5.07	0.26 (0.02)	1.20 (1.02)
Vascular disorders	10047065	112	0.36 (0.30–0.43)	0.37 (0.30–0.44)	125.70	-1.44 (-1.71)	0.37 (0.30)
Ear and labyrinth disorders	10013993	106	1.64 (1.36–1.99)	1.64 (1.35–1.98)	26.33	0.70 (0.42)	1.64 (1.35)
Renal and urinary disorders	10038359	78	0.27 (0.22–0.34)	0.27 (0.22–0.34)	153.76	-1.86 (-2.18)	0.27 (0.22)
Infections and infestations	10021881	73	0.09 (0.07–0.11)	0.09 (0.07–0.12)	681.13	-3.41 (-3.74)	0.09 (0.07)
Surgical and medical procedures	10042613	66	0.33 (0.26–0.42)	0.33 (0.26–0.42)	89.16	-1.57 (-1.92)	0.33 (0.26)
Social circumstances	10041244	60	0.92 (0.71–1.19)	0.92 (0.72–1.19)	0.40	-0.12 (-0.49)	0.92 (0.72)
Immune system disorders	10021428	55	0.32 (0.25–0.42)	0.33 (0.25–0.43)	77.22	-1.60 (-1.98)	0.33 (0.25)
Product issues	10077536	54	0.22 (0.17–0.28)	0.22 (0.17–0.29)	153.30	-2.17 (-2.56)	0.22 (0.17)
Neoplasms benign, malignant and unspecified (incl cysts and polyps)	10029104	17	0.04 (0.02–0.06)	0.04 (0.02–0.06)	402.09	-4.56 (-5.24)	0.04 (0.02)
Blood and lymphatic system disorders	10005329	16	0.07 (0.04–0.11)	0.07 (0.04–0.11)	213.98	-3.83 (-4.53)	0.07 (0.04)
Hepatobiliary disorders	10019805	15	0.12 (0.07–0.20)	0.12 (0.07–0.20)	97.97	-2.98 (-3.70)	0.12 (0.07)
Endocrine disorders	10014698	11	0.29 (0.16–0.53)	0.29 (0.16–0.53)	18.61	-1.67 (-2.51)	0.29 (0.16)
Pregnancy, puerperium and perinatal conditions	10036585	8	0.13 (0.07–0.26)	0.13 (0.07–0.27)	45.57	-2.77 (-3.73)	0.13 (0.07)
Congenital, familial and genetic disorders	10010331	1	0.02 (0.00–0.16)	0.02 (0.00–0.16)	41.40	-4.47 (-6.51)	0.02 (0.00)

Based on the frequency of occurrence and EBGM values of PTs, the top 30 are listed in Tables 5 and 6. Among these, Diarrhoea, Nausea, Insomnia, Anxiety, Dizziness, Agitation, Tremor, Weight increased had higher incidence rates, aligning with the label records. Simultaneously, strong signals for new potential AEs were identified, including sleep disorders like Sleep paralysis, Hypnagogic hallucination, Rapid eye movements sleep abnormal, Sleep terror, Terminal insomnia, Tachyphrenia; sexual dysfunctions such as Female orgasmic disorder, Orgasm abnormal, Disturbance in sexual arousal, Spontaneous penile erection, Anorgasmia, Sexual dysfunction, Ejaculation delayed; and other symptoms and injuries like Electric shock sensation, Violence-related symptom, Gun shot wound.

**Table 5:** Top 30 PTs by report numbers for vilazodone AEs

SOC	PTs	Case Reports	ROR (95% CI)	PRR (95% CI)	χ2	IC (IC025)	EBGM (EBGM05)
Injury, poisoning and procedural complications	Off label use	929	4.73 (4.42–5.05)	4.49 (4.22–4.78)	2555.15	2.16 (2.06)	4.49 (4.20)
Gastrointestinal disorders	Diarrhoea	633	4.19 (3.87–4.54)	4.05 (3.76–4.38)	1470.19	2.01 (1.89)	4.05 (3.74)
Gastrointestinal disorders	Nausea	514	2.83 (2.59–3.09)	2.77 (2.54–3.01)	586.24	1.46 (1.33)	2.76 (2.53)
Psychiatric disorders	Insomnia	505	8.17 (7.48–8.93)	7.93 (7.27–8.64)	3061.21	2.96 (2.83)	7.91 (7.23)
Psychiatric disorders	Anxiety	365	5.32 (4.79–5.90)	5.21 (4.71–5.77)	1245.05	2.36 (2.21)	5.20 (4.69)
Nervous system disorders	Dizziness	310	2.67 (2.39–2.99)	2.64 (2.36–2.94)	317.29	1.39 (1.23)	2.64 (2.36)
Nervous system disorders	Paraesthesia	266	7.06 (6.26–7.98)	6.96 (6.17–7.84)	1356.55	2.76 (2.58)	6.94 (6.15)
General disorders and administration site conditions	Feeling abnormal	259	4.28 (3.78–4.83)	4.22 (3.74–4.76)	637.54	2.06 (1.88)	4.21 (3.73)
Psychiatric disorders	Suicidal ideation	248	12.71 (11.20–14.41)	12.51 (11.06–14.16)	2618.68	3.57 (3.39)	12.46 (10.99)
Psychiatric disorders	Depression	243	4.63 (4.08–5.25)	4.57 (4.03–5.17)	678.51	2.17 (1.98)	4.56 (4.02)
Psychiatric disorders	Hallucination	172	10.73 (9.23–12.47)	10.61 (9.15–12.32)	1493.91	3.33 (3.10)	10.58 (9.10)
Psychiatric disorders	Agitation	164	10.13 (8.69–11.82)	10.03 (8.61–11.69)	1330.55	3.25 (3.02)	10.00 (8.57)
Nervous system disorders	Tremor	151	3.93 (3.35–4.61)	3.90 (3.33–4.57)	325.95	1.93 (1.70)	3.90 (3.32)
Investigations	Weight increased	142	2.76 (2.34–3.26)	2.74 (2.33–3.23)	157.59	1.44 (1.19)	2.74 (2.32)
Psychiatric disorders	Abnormal dreams	141	25.33 (21.45–29.93)	25.10 (21.28–29.61)	3236.04	4.41 (4.17)	24.89 (21.07)
Nervous system disorders	Somnolence	140	2.99 (2.53–3.53)	2.97 (2.52–3.50)	183.32	1.55 (1.31)	2.97 (2.51)
Psychiatric disorders	Nightmare	137	17.86 (15.09–21.14)	17.71 (14.98–20.93)	2147.30	3.97 (3.73)	17.60 (14.87)
Psychiatric disorders	Irritability	131	9.23 (7.77–10.97)	9.16 (7.72–10.86)	949.90	3.10 (2.85)	9.13 (7.69)
General disorders and administration site conditions	Crying	121	14.49 (12.11–17.34)	14.38 (12.04–17.18)	1499.70	3.69 (3.43)	14.31 (11.96)
Cardiac disorders	Palpitations	108	3.97 (3.28–4.80)	3.95 (3.27–4.76)	237.79	1.94 (1.66)	3.94 (3.26)
Skin and subcutaneous tissue disorders	Hyperhidrosis	104	3.46 (2.85–4.20)	3.44 (2.84–4.17)	180.50	1.75 (1.47)	3.44 (2.84)
Psychiatric disorders	Confusional state	104	2.80 (2.31–3.40)	2.79 (2.30–3.38)	119.47	1.45 (1.17)	2.79 (2.30)
Psychiatric disorders	Anger	97	12.50 (10.23–15.27)	12.42 (10.18–15.15)	1014.91	3.47 (3.18)	12.37 (10.13)
Psychiatric disorders	Panic attack	88	10.68 (8.65–13.17)	10.62 (8.62–13.08)	764.36	3.26 (2.95)	10.58 (8.58)
Eye disorders	Vision blurred	85	2.73 (2.20–3.37)	2.72 (2.20–3.36)	92.25	1.41 (1.10)	2.71 (2.19)
Nervous system disorders	Serotonin syndrome	81	19.96 (16.04–24.86)	19.86 (15.97–24.70)	1441.26	4.01 (3.68)	19.73 (15.85)
Psychiatric disorders	Mania	77	21.90 (17.49–27.41)	21.79 (17.42–27.25)	1516.03	4.10 (3.77)	21.63 (17.28)
Psychiatric disorders	Aggression	69	6.52 (5.15–8.27)	6.50 (5.13–8.22)	320.45	2.59 (2.24)	6.49 (5.12)
Musculoskeletal and connective tissue disorders	Muscle twitching	61	11.43 (8.88–14.70)	11.38 (8.86–14.63)	575.68	3.28 (2.91)	11.34 (8.82)
General disorders and administration site conditions	Feeling jittery	58	13.43 (10.37–17.40)	13.38 (10.35–17.32)	661.77	3.46 (3.09)	13.33 (10.29)

**Table 6:** The top signal strength of AEs of vilazodone ranked by EBGM at the PTs level

SOC	PTs	Case Reports	ROR (95% CI)	PRR (95% CI)	χ2	IC (IC025)	EBGM (EBGM05)
Nervous system disorders	Sleep paralysis	55	157.34 (119.89–206.50)	156.76 (119.56–205.54)	8072.54	5.35 (4.96)	148.71 (113.31)
Psychiatric disorders	Hypnagogic hallucination	8	118.66 (58.50–240.70)	118.59 (58.49–240.48)	895.88	3.07 (2.09)	113.94 (56.17)
Psychiatric disorders	Female orgasmic disorder	4	54.28 (20.18–145.97)	54.26 (20.18–145.90)	205.25	2.22 (0.91)	53.28 (19.81)
Psychiatric disorders	Rapid eye movements sleep abnormal	5	51.56 (21.29–124.86)	51.54 (21.29–124.78)	243.43	2.45 (1.26)	50.65 (20.91)
Injury, poisoning and procedural complications	Discontinued product administered	3	46.90 (14.99–146.78)	46.89 (14.99–146.71)	132.57	1.91 (0.45)	46.15 (14.75)
Nervous system disorders	Cold-stimulus headache	3	37.52 (12.01–117.21)	37.51 (12.01–117.16)	105.24	1.89 (0.43)	37.04 (11.86)
Psychiatric disorders	Sleep terror	33	34.97 (24.80–49.31)	34.89 (24.76–49.16)	1073.41	4.12 (3.62)	34.49 (24.46)
Psychiatric disorders	Orgasm abnormal	6	34.46 (15.40–77.07)	34.44 (15.40–77.02)	192.52	2.57 (1.47)	34.05 (15.22)
Nervous system disorders	Electric shock sensation	15	33.68 (20.24–56.05)	33.65 (20.23–55.97)	469.71	3.46 (2.74)	33.27 (19.99)
Psychiatric disorders	Violence-related symptom	10	28.72 (15.40–53.55)	28.70 (15.40–53.50)	264.73	3.02 (2.15)	28.43 (15.25)
Psychiatric disorders	Terminal insomnia	9	26.95 (13.98–51.96)	26.93 (13.97–51.91)	222.66	2.90 (1.98)	26.69 (13.84)
Psychiatric disorders	Tachyphrenia	23	25.89 (17.17–39.04)	25.85 (17.15–38.96)	544.54	3.66 (3.07)	25.63 (16.99)
Injury, poisoning and procedural complications	Gun shot wound	7	25.66 (12.19–54.01)	25.64 (12.19–53.96)	164.33	2.65 (1.62)	25.43 (12.08)
Psychiatric disorders	Abnormal dreams	141	25.33 (21.45–29.93)	25.10 (21.28–29.61)	3236.04	4.41 (4.17)	24.89 (21.07)
Reproductive system and breast disorders	Ejaculation delayed	3	22.89 (7.35–71.30)	22.89 (7.35–71.27)	62.29	1.82 (0.37)	22.71 (7.29)
Psychiatric disorders	Disturbance in sexual arousal	5	22.23 (9.22–53.60)	22.22 (9.22–53.57)	100.57	2.29 (1.11)	22.06 (9.15)
Reproductive system and breast disorders	Spontaneous penile erection	3	21.90 (7.03–68.21)	21.90 (7.03–68.18)	59.38	1.81 (0.36)	21.74 (6.98)
Psychiatric disorders	Mania	77	21.90 (17.49–27.41)	21.79 (17.42–27.25)	1516.03	4.10 (3.77)	21.63 (17.28)
Psychiatric disorders	Morbid thoughts	5	21.25 (8.81–51.22)	21.24 (8.81–51.19)	95.73	2.28 (1.09)	21.09 (8.75)
Psychiatric disorders	Anorgasmia	12	20.67 (11.71–36.48)	20.65 (11.71–36.43)	222.82	3.04 (2.23)	20.51 (11.62)
Nervous system disorders	Serotonin syndrome	81	19.96 (16.04–24.86)	19.86 (15.97–24.70)	1441.26	4.01 (3.68)	19.73 (15.85)
Psychiatric disorders	Hypomania	16	19.60 (11.98–32.06)	19.58 (11.98–32.01)	280.21	3.22 (2.52)	19.45 (11.90)
Injury, poisoning and procedural complications	Drug dose titration not performed	8	18.30 (9.13–36.68)	18.29 (9.13–36.64)	129.92	2.64 (1.68)	18.18 (9.07)
Psychiatric disorders	Nightmare	137	17.86 (15.09–21.14)	17.71 (14.98–20.93)	2147.30	3.97 (3.73)	17.60 (14.87)
Reproductive system and breast disorders	Sexual dysfunction	47	17.62 (13.22–23.49)	17.57 (13.20–23.39)	730.15	3.70 (3.28)	17.47 (13.11)
Psychiatric disorders	Hostility	8	15.83 (7.90–31.72)	15.82 (7.90–31.70)	110.49	2.58 (1.61)	15.74 (7.86)
General disorders and administration site conditions	Crying	121	14.49 (12.11–17.34)	14.38 (12.04–17.18)	1499.70	3.69 (3.43)	14.31 (11.96)
Psychiatric disorders	Obsessive thoughts	5	13.41 (5.57–32.28)	13.40 (5.57–32.26)	57.11	2.13 (0.94)	13.34 (5.54)
General disorders and administration site conditions	Feeling jittery	58	13.43 (10.37–17.40)	13.38 (10.35–17.32)	661.77	3.46 (3.09)	13.33 (10.29)
Injury, poisoning and procedural complications	Drug titration error	8	12.77 (6.38–25.59)	12.77 (6.38–25.57)	86.39	2.47 (1.50)	12.72 (6.35)

## Discussion

Vilazodone, as a novel selective SSRI, has attracted attention for its prominent agonistic effects on the 5-HT1A receptor and a longer half-life, promising to further improve the clinical cure rates for MDD and enhance patient medication adherence. However, like all medications, Vilazodone's use is associated with the risk of AEs, a crucial factor for clinicians and patients to consider when contemplating pharmacotherapy [16]. Therefore, with the continuous development and iteration of antidepressants, a comprehensive assessment of their efficacy and safety becomes a key task in the treatment of depression.

The study revealed specific gender and age tendencies, with 65.40% of the reports coming from females [17]. This may reflect the prevalence of antidepressant use among women and their proactivity in reporting drug adverse reactions. In terms of age, the highest reports were in the 45 to 65 age group, indicating that middle-aged and older adults might be the primary users of Vilazodone. The fluctuating decrease in report numbers since the peak in 2013 might be related to the heightened initial attention following the drug's release and subsequent reduction in monitoring. The majority of reports were submitted by consumers, predominantly in the United States, highlighting the significant role of patients in drug safety monitoring and geographical usage differences. The most common severe AEs included hospitalization, death, and disability, underscoring the potential serious risks of Vilazodone. Most AEs occurred within 30 days of medication use, emphasizing the critical importance of monitoring during the initial treatment phase. These findings contribute to understanding Vilazodone's safety profile, hold significant implications for improving clinical practice and patient management, and underscore the necessity for ongoing drug safety monitoring and individualized treatment approaches.

In the process of risk signal mining for AEs associated with Vilazodone, this study identified a range of AEs related to the drug's use. These included both known AEs already documented in the drug's label and new, potential AEs. The most common AEs included Psychiatric disorders, Nervous system disorders, Gastrointestinal disorders, and General disorders and administration site conditions, aligning with Vilazodone’s label information. Events such as Diarrhoea, Nausea, and Insomnia were noted for their higher incidence rates.

New Potential AEs were also identified. Sleep Disorders: Including Sleep paralysis, Hypnagogic hallucination, Rapid eye movements sleep abnormal, Sleep terror, Terminal insomnia, Tachyphrenia, these could stem from Vilazodone’s modulation of neurotransmitters and the sleep cycle. As a selective serotonin reuptake inhibitor and 5-HT1A receptor agonist, Vilazodone impacts the levels of serotonin and other neurotransmitters like dopamine and norepinephrine [18, 19]. This influence might lead to sleep paralysis (due to an imbalance in REM sleep regulation), Hypnagogic hallucination, and Tachyphrenia (due to alterations in cognitive processes and perception). Furthermore, Vilazodone’s effect on sleep cycles may result in Rapid Eye Movements Sleep Abnormal, Sleep Terror, and Terminal Insomnia, potentially originating from the drug’s adjustment of sleep architecture and depth [20]. The interplay of these mechanisms, especially Vilazodone’s impact on various neurotransmitter systems, could lead to complex sleep disturbances, reflecting the drug’s extensive influence on brain function and sleep regulation.

Sexual Dysfunction: Including Female orgasmic disorder, Orgasm abnormal, Disturbance in sexual arousal, Spontaneous penile erection, Anorgasmia, Sexual dysfunction, Ejaculation delayed. The sexual dysfunctions caused by Vilazodone could primarily be attributed to two mechanisms: altered neurotransmitter balance and the drug’s physiological actions. Firstly, as a selective SSRI, Vilazodone increases brain serotonin levels, which may indirectly affect the release of sex hormones and sexual function [21, 22]. Elevated serotonin levels could lead to reduced libido, sexual arousal disorders, thereby causing Female orgasmic disorder, Orgasm abnormal, Disturbance in sexual arousal, and similar issues. Secondly, Vilazodone’s impact on physiological responses, such as affecting blood flow and neural responses [23], might lead to Spontaneous penile erection, Anorgasmia, Ejaculation delayed, and other symptoms. These effects reflect Vilazodone’s extensive action on sex hormones and the nervous system, potentially leading to complex changes in sexual function.

The Electric shock sensation, Violence-related symptom, and Gun shot wound possibly linked to Vilazodone may be attributed to the drug's impact on neurotransmitter balance and hypersensitivity of the nervous system [24]. These symptoms underscore the need for cautious monitoring of patient reactions when using Vilazodone, particularly during dose adjustments or withdrawal periods.

This study is subject to several limitations. Firstly, reliance on spontaneous reporting to the FAERS database may introduce reporting biases and underreporting of adverse events. The database does not provide comprehensive data on the genetic or ethnic backgrounds of individuals, which could significantly influence the pharmacodynamics and pharmacokinetics of vilazodone. Additionally, this study primarily focuses on adverse event reports submitted to the FAERS database, which predominantly includes data from the United States. Therefore, our findings may not fully represent the adverse event profile of vilazodone in other populations, including China. Without including other antidepressants as controls, it is difficult to determine whether the adverse reactions found also exist in existing drugs, or if they are novel risks specific to Vilazodone. Future research should aim to include control groups of commonly used antidepressants like SSRIs and SNRIs to perform comparative assessments across multiple drugs. By analyzing genetic, ethnic, and regional variations in future studies, and by calculating report ratios and assessing signal strength differences, it would be possible to more clearly differentiate Vilazodone's unique spectrum of adverse reactions and enhance the generalizability of these findings to other populations.

## Conclusion

This study, through an in-depth analysis of data from the FAERS database, has revealed the safety characteristics and potential AE risks of Vilazodone in clinical use. As a novel antidepressant, Vilazodone has demonstrated efficacy in treating MDD, but our findings emphasize the importance of a thorough understanding of its safety profile. The analysis revealed that reports related to Vilazodone use predominantly involved women and patients in the 45 to 65 age group, which might reflect the sensitivity of these specific demographics to the drug. The main categories of AEs included Psychiatric disorders, Nervous system disorders, and Gastrointestinal disorders, aligning with the drug’s label information. Notably, this study also identified new potential AEs, such as sleep disorders and sexual dysfunctions, suggesting that clinicians should be vigilant in monitoring and managing these potential risks when prescribing Vilazodone.

## Data Availability

The dataset generated during and analyzed during the current study are available from the corresponding author on reasonable request.
